# On the Reunion of Wounds of the Spinal Cord, with Restoration of Its Lost Functions

**Published:** 1851-11

**Authors:** 


### ANATOMY AND PHYSIOLOGY.

*On the Reunion of Wounds of the Spinal Cord, with Restoration of its lost Functions.* By M. Brown-Sequard.—During the last three years, M. Brown-Sequard has made a considerable number of experiments, with a view of determining the degree of reparative power which exists in the Spinal Cord; the results of which are very remarkable. The following is one of the most striking:—The spinal cord of a Pigeon was *entirely divided* between the 5th and 6th dorsal vertebræ; and the operation was followed by complete paralysis of the posterior part of the body, as regarded sensibility and voluntary movement. At the end of three months, voluntary movements began to show themselves, in the midst of reflex actions; and sensibility also reappeared. These powers gradually augmented; and six months after the operation, the bird could stand for some minutes, but fell if it attempted to walk. In the course of the seventh month it began to walk, but unsteadily, helping itself by its wings. By the end of the eighth month, it could walk slowly without support; but if it attempted to walk fast, it fell over, unless it supported itself by its wings. Twelve months after the operation, it could run; and when the account of the case was drawn up, fifteen months after the section had been made, its progression seemed in all respects normal, save that a certain degree of stiffness remained in its gait.

In several Guinea-pigs, in which the section had only been made through one half of the spinal cord, an incomplete return of voluntary power was observed within seven or eight months after the operation. In the case of one Guinea-pig, which had been subjected to this operation a year before, and in which sensibility appeared to have been completely restored, and voluntary movement less completely, a careful examination was made of the injured part. It was found that the section had traversed both the posterior columns, as well as the anterior and lateral columns, and a portion of the grey substance on the right side; all of which parts exhibited a sort of contraction, the continuity of the divided parts being re-established by a whitish cicatrix. On examining the substance of this cicatrix, it was found to be in great part made up of fibres of areolar tissue, the direction of which was transverse or oblique; but these were crossed by great numbers of nerve-fibres running in a longitudinal direction, which exhibited a double contour, and were uninterruptedly continuous through the whole extent of the cicatrix. Among these were scattered some ganglionic corpuscles. A like reproduction of nerve-fibres in the cicatrix of the spinal cord, has been substantiated by M. Brown-Sequard in two other cases.—*Brit. and For. Review, Oct.* 1851, *from Gazette Médicale.*

